# Spatio-Temporal Water Hyacinth Monitoring in the Lower Mondego (Portugal) Using Remote Sensing Data

**DOI:** 10.3390/plants11243465

**Published:** 2022-12-10

**Authors:** Luís Pádua, Lia Duarte, Ana M. Antão-Geraldes, Joaquim J. Sousa, João Paulo Castro

**Affiliations:** 1 Centre for the Research and Technology of Agro-Environmental and Biological Sciences, University of Trás-os-Montes e Alto Douro, 5000-801 Vila Real, Portugal; 2 Institute for Innovation, Capacity Building and Sustainability of Agri-Food Production, University of Trás-os-Montes e Alto Douro, 5000-801 Vila Real, Portugal; 3 Institute of Earth Sciences, FCUP Pole, Rua do Campo Alegre, 4169-007 Porto, Portugal; 4 Department of Geosciences, Environment and Spatial Planning, FCUP, 4169-007 Porto, Portugal; 5 Centro de Investigação de Montanha (CIMO), Instituto Politécnico de Bragança Campus de Santa Apolónia, 5300-253 Bragança, Portugal; 6 Laboratório Associado para a Sustentabilidade e Tecnologia em Regiões de Montanha (SusTEC), Instituto Politécnico de Bragança, Campus de Santa Apolónia, 5300-253 Bragança, Portugal; 7 Engineering Department, School of Science and Technology, University of Trás-os-Montes e Alto Douro, 5000-801 Vila Real, Portugal; 8 Centre for Robotics in Industry and Intelligent Systems (CRIIS), INESC Technology and Science (INESC-TEC), 4200-465 Porto, Portugal

**Keywords:** satellite, invasive species, normalized difference vegetation index, remote sensing, geographical information systems

## Abstract

Monitoring invasive plant species is a crucial task to assess their presence in affected ecosystems. However, it is a laborious and complex task as it requires vast surface areas, with difficult access, to be surveyed. Remotely sensed data can be a great contribution to such operations, especially for clearly visible and predominant species. In the scope of this study, water hyacinth (*Eichhornia crassipes*) was monitored in the Lower Mondego region (Portugal). For this purpose, Sentinel-2 satellite data were explored enabling us to follow spatial patterns in three water channels from 2018 to 2021. By applying a straightforward and effective methodology, it was possible to estimate areas that could contain water hyacinth and to obtain the total surface area occupied by this invasive species. The normalized difference vegetation index (NDVI) was used for this purpose. It was verified that the occupation of this invasive species over the study area exponentially increases from May to October. However, this increase was not verified in 2021, which could be a consequence of the adopted mitigation measures. To provide the results of this study, the methodology was applied through a semi-automatic geographic information system (GIS) application. This tool enables researchers and ecologists to apply the same approach in monitoring water hyacinth or any other invasive plant species in similar or different contexts. This methodology proved to be more effective than machine learning approaches when applied to multispectral data acquired with an unmanned aerial vehicle. In fact, a global accuracy greater than 97% was achieved using the NDVI-based approach, versus 93% when using the machine learning approach (above 93%).

## 1. Introduction

Water hyacinth (*Eichhornia crassipes*) is a free-floating macrophyte native to the Amazon basin and adapted to lentic habitats. Its high ornamental value made it spread globally since the late nineteenth century. Nowadays, water hyacinth is present in lakes, reservoirs, ponds, irrigation ditches, and the final sections of rivers on all continents except Antarctica. It is in the top 100 of the most exotic and considered one of the most aggressive and predominant invasive species in the world [1]. Indeed, *Eichhornia crassipes* reproduction is defined by asexual reproduction and also by the production of a high number of seeds (via sexual reproduction)—a single inflorescence with 20 flowers can produce about 3000 seeds, which can remain viable for about 20 years—providing a competitive advantage over other native macrophytes [2,3] in invaded ecosystems. Furthermore, the vegetative reproduction originates from plant fragments, which can survive over winter and be disseminated by winds and currents, creating new invasion foci [4]. Therefore, in invaded areas, this plant creates extensive mats covering the water surface, changing the aquatic ecosystem, and, in certain cases, leading to the elimination of native aquatic macrophytes. Consequently, these changes cause a decrease in phytoplankton, zooplankton, macroinvertebrate, and fish diversity, due to degradation of the habitat induced by decreasing light input and dissolved oxygen, increasing turbidity, and reducing water quality. It also provides several disadvantages such as the obstruction of irrigation and navigation canals, which affects water usage and activities such as navigation, fisheries, agriculture, tourism, and hydroelectric power generation by blocking turbines and decreasing agricultural productivity (e.g., invasion of rice fields) and grazing land (increasing livestock production cost) [2,5,6,7,8,9].

Low nutrient availability, temperatures lower than 10 °C, and elevated salinity levels are the most significant limiting factors for water hyacinth expansion. Therefore, the aquatic ecosystems located at latitudes between 40° N and 40° S are the most vulnerable to invasion. In Europe, the southern regions, such as Portugal, Spain, Italy, and France (Corsica island) present the most susceptibility to water hyacinth invasions [2]. However, in a climate change scenario, it is expected that invasive capacity will extend to other regions of Europe [2,5]. In Portugal, the water hyacinth’s first sighting occurred in 1939 [10]. Nowadays, it is spread across the various regions of the country, including Terceira Island (Azores Archipelago). Some of these invasions deserve special attention, such as the cases of the Paúl do Boquilobo (biosphere reserve), irrigation channels, rice fields in the Sado and Sorraia sub-basins (Tagus Basin) [11,12], and Pateira de Fermentelos, one of the largest freshwater lagoons of the Iberian Peninsula [13]. In the Guadiana Basin it covers more than 200 ha, invading Alqueva Reservoir [2,4]. Based on the foregoing, it can be concluded that water hyacinth is extremely difficult to eradicate once established [6]. The combination of all these factors led to the creation of a European regulation (EU Regulation No. 1143/2014) stating that this species cannot be introduced, maintained, reproduced, or commercialized in the European space. In the specific case of Portugal, legal restrictions on the use and dissemination of water hyacinth were introduced even before the European legislation (Decree-Law no. 565/99 of 21 December, revised by Decree-Law no. 92/2019, of 10 July). More recently, the National Assembly Resolution no. 13/2020 recommends creating a national control plan for this species to minimize economic costs and ecological impacts. Therefore, to implement the control plan, a better understanding of water hyacinth spatiotemporal dynamics is needed.

Remote sensing and specifically, satellite imagery data analysis, are added-value tools to investigate and monitor water hyacinth invasion dynamics on a large scale. Indeed, and despite the low spatial resolution of satellite images when compared to high spatial resolution images obtained by unmanned aerial vehicles (UAVs), the correlation between the two types of data is high, making satellite images, namely Sentinel-2 imagery, appropriate tools for spatiotemporal studies [14]. Thus, it also provides helpful information, allowing the implementation of appropriate management strategies to control and prevent invasions, thus promoting the early eradication of this invasive macrophyte.

Decision making in the management and control of water-hyacinth-affected ecosystems needs constant local monitoring to assess the invasion extent [15]. The effectiveness of using satellite imagery to monitor the spread of water hyacinth over aquatic ecosystems has already been proven [16,17]. A wide range of studies can be found in the literature that used multispectral satellite imagery data from different platforms, such as Landsat medium-resolution data (30 m spatial resolution). Dube et al. [18] monitored water hyacinth presence in Lake Chivero (Zimbabwe) using Landsat 8 data classified with discriminant analysis (DA) and partial least squares discriminant analysis (PLS-DA). An overall accuracy of 95% was obtained, with DA outperforming PLS-DA. Moreover, from the classified water hyacinth extent, the authors were able to classify different age groups (old, intermediate, and young). In Dube et al. [15], water hyacinth classification, comparing Landsat 8 Operational Land Imager (OLI) and Landsat 7 Enhanced Thematic Mapper (ETM) data, was performed, with Landsat 8 OLI data reaching a higher overall classification accuracy. Mukarugwiro et al. [19] used Landsat 8 data to map water hyacinth spatial distribution in Rwandan waterbodies. The authors classified different land-cover types and evaluated two machine learning techniques: random forest (RF) and support vector machines (SVM), with RF reaching the best performance. By applying the same approach, Mukarugwiro et al. [20] mapped water hyacinth spatiotemporal variation using Landsat satellite imagery from 1989, 2002, and 2017. With temporal data it was possible to estimate the water hyacinth coverage in each period and its increase. Commercial platforms as WorldView-2 (0.46 m and 1.84 m of spatial resolution for panchromatic and multispectral data, respectively) were also used [21,22]. John and Kavya [21] used WorldView-2 to map aquatic macrophyte communities, including water hyacinth, in the Vembanad estuary (India), using unsupervised classification based on an iterative self-organizing data analysis technique (ISODATA) algorithm. Damtie, Mengistu and Meshesha [23] monitored Lake Tana (Ethiopia), using four images, one for each season, from Sentinel-2 (10 m spatial resolution), observing the effect of this invasive plant on water loss by evapotranspiration increase and mapping its presence using a maximum likelihood classifier. Using the same classification method, Damtie and Mengistsu [24] evaluated the impacts of water hyacinth on land use and land cover in northeastern Lake Tana. The authors used data before water hyacinth presence (2010) from Landsat 5 (30 m spatial resolution) comparing it with 2019 data from Sentinel-2 and it was possible to map not only its proliferation but also to observe that water, agricultural land, and bare land areas suffered an area coverage reduction in favor of water hyacinth. Asmare et al. [25], evaluated a 5-year period (December 2013, 2015, and 2017) in Lake Tana using Landsat 8 data and a decision tree classifier. The amount of coverage increased from 112 to 1512 ha. Janssens et al. [22] explored the use of Sentinel-2 data to monitor water hyacinth invasion severity in the Saigon river (Vietnam). It was possible to map the seasonal dynamics of water hyacinth coverage from 2018 to 2020 by using a Naïve Bayes classifier. To benefit from the different time series satellite data collected through time, Ongore et al. [26] monitored the spatiotemporal dynamics of water hyacinth in Lake Victoria (Kenya) using Landsat 7 (January 2014 to July 2015), Landsat 8 (July 2015 to December 2016) and Sentinel-2 (January 2017 to December 2017) data.

Thamaga and Dube [16] stated that there is still a need to explore the applicability of non-commercial new-generation spatial sensors to monitor water hyacinth in small reservoirs (such as Sentinel-2). The use of such data will make it possible to understand the spatiotemporal evolution of water hyacinth and the development of operational monitoring tools, making control and eradication programs effective and robust [18]. The European Space Agency (ESA) Sentinel-2 satellite provides time series with frequent temporal coverage, at high spectral resolution and at no cost to the user, allowing for the monitoring of river stretches and narrower channels. In this context, Ghoussein et al. [27] performed multi-temporal mapping of water hyacinth in the Al Kabir river (Lebanon), assessing the potential of Sentinel-2 data for tracking invasive water hyacinth in a river branch between 2015 and 2020. Gerardo and Lima [28] assessed the use of Sentinel-2 data to map water hyacinth in a small water course from the Mondego river (Portugal) between 2017 and 2021. These studies made use of vegetation indices such as normalized difference vegetation index (NDVI) [29], normalized difference water index (NDWI) [30], and soil adjusted vegetation index (SAVI) [31]. More recently, high spatial-resolution multispectral data acquired using a UAV was also explored to detect water hyacinth growth along with Sentinel-2 data through the use of machine learning classifiers [32], allowing users to map water hyacinth in periods where Sentinel-2 data are not available. Despite the great advances provided using remote sensing data for the monitoring and management of invasive plants in general, and of water hyacinth in particular, most of the studies carried out are based on complex methodologies. This makes their implementation difficult by the technicians who directly intervene in its management, normally without the background knowledge that allows them to follow these methods. After the phase in which the effectiveness and efficiency of remote sensing data in this type of application was proven, it is also important to develop new and straightforward methodologies that allow, at the same time, the creation of tools that can be used by technicians with different levels of training, but who directly deal with these issues. With this study, it is intended to advance towards that direction. Water hyacinth dispersion dynamics are addressed in a multi-temporal perspective by using Sentinel-2 multispectral data to monitor small water channels in the Lower Mondego (Portugal), which is severely affected by this invasive species and where mitigation/containment measures are being taken. The employed methodology intends to create a straightforward yet effective pipeline based on vegetation index thresholding to compute water hyacinth presence within the water channels intended to be monitored. A geographic information system (GIS) tool that integrates the methodology to detect the invasive species was developed by the research team. The tool is free and open source, providing the advantage to be adapted to other species and other contexts. It will thus allow ecologists and researchers with little or no knowledge of GIS to use remote sensing data to perform such analysis, enabling to improve their efforts to reduce this invasive species.

## 2. Materials and Methods

### 2.1. Study Area Characterization

**Figure 1:**
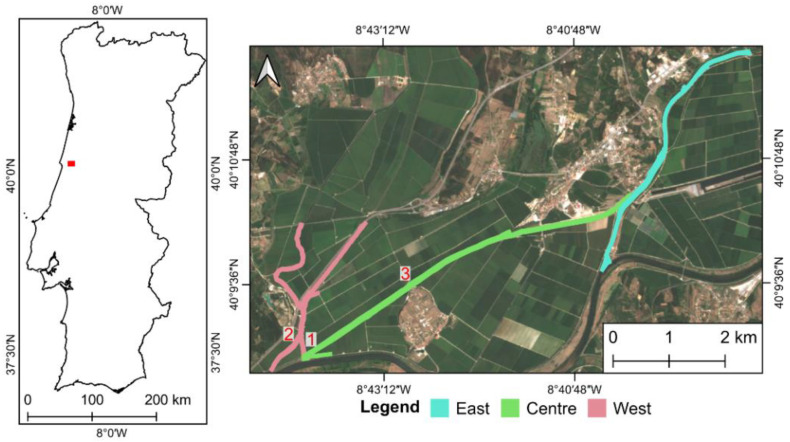
Study area overview: its location within mainland Portugal (red polygon), the extension of the analyzed water channels, and the three locations surveyed with the unmanned aerial vehicle.

The area analyzed in this study is in central Portugal, within the Mondego River basin, between the cities of Coimbra and Figueira da Foz, a region known as Lower Mondego (Baixo Mondego in Portuguese). It is mostly composed of rice fields and three channels were selected to be monitored. These locations have diversion channels with potential accumulation of water hyacinth (Figure 1). A total area of approximately 46 ha was monitored. The west channel has a size of 10.7 ha (23.2% of the total analyzed area), which has two derivation points, one to the Mondego River main water course and another to the central channel. The central channel, with 25 ha, represents 54.2% of the studied hydric surface which derives to the Mondego River at the end. The east channel has 10.4 ha, represents 22.6% of the analyzed area, and flows to the derivation point of the central channel.

### 2.2. Remote Sensing Data

Two types of remote sensing data were used to carry out this study: Sentinel-2 Multispectral Instrument (MSI) imagery and UAV multispectral imagery. The former group of images constitutes the core of this study, while the latter were used for validation purposes. The spectral data products provided by the MSI range from the visible to the short-wave infrared parts of the electromagnetic spectrum. Thirteen spectral bands (B) are available, in total, with different spatial resolutions central wavelengths: (1) with 10 m—B2 (490 nm), B3 (560 nm), B4 (665 nm) e B8 (842 nm); (2) with 20 m—B5 (705 nm), B6 (740 nm), B7 (783 nm), B8a (865 nm), B11 (1610 nm) e B12 (2190 nm); and (3) with 60 m—B1 (443 nm), B9 (940 nm) e B10 (1375 nm). The acquired data were projected onto a 100 km × 100 km Universal Transverse Mercator (UTM) grid.

The data were downloaded using the Mundi Web Services platform. The search query respected the following conditions: Level 2A data products, corresponding to Bottom-Of-Atmosphere (BOA) reflectance; containing a cloud cover percentage below 10%; and between May 2018 and October 2021. Thus, all 10 m spatial resolution bands were downloaded (B2, B3, B4, B8), as well as a true color image (TCI) representation of each period. Table 1 shows the dates selected for the study. There were periods in which the conditions of the available data did not meet the search criteria, mainly due to a high cloud cover percentage over the study area. It should be noted that for this specific study, only data from the same grid (T29TNE) were used.

UAV-based data were obtained on 21 July 2021 in the locations numbered in Figure 1. They were acquired using a Matrice 300 RTK (DJI, Shenzhen, China) with a RedEdge-MX sensor (MicaSense, Inc., Seattle, DC, USA) coupled to it. This way, high-spatial-resolution (approximately 0.07 m) data from blue (475 nm), green (560 nm), red (668 nm), red edge (717 nm), and near infrared (NIR) (842 nm) spectral bands were available. For more details on the UAV data acquisition and processing please refer to Pádua et al. [32].

### 2.3. Water Hyacinth Spatio-Temporal Monitoring

The methodology implemented consists of a logical sequence of steps in order to obtain the spatial distribution of water hyacinth. If applied in a temporal perspective, this methodology allows measurement of the temporal evolution of the area covered by water hyacinth and its spatial distribution. In this way, it will be possible to simplify the observation of seasonal dynamics in the study area and estimate which areas are most affected.

From the remotely sensed data, vegetation indices were computed, enabling us to highlight vegetation by considering differences obtained from arithmetic operations towards the available spectral bands. Usually, vegetation indices present higher values for healthy vegetation in comparison to non-vegetation elements, such as soil, dry vegetation, human-made infrastructures, and water. Since water hyacinth is mainly present in aquatic environments it is important to circumscribe the area to allocate the specimens to areas that meet the characteristics. Therefore, it is necessary to provide the polygons with locations that possibly contain water hyacinth. In the case of this study, a set of polygons delimitating the water channels was used in the analysis.

After identifying the areas that can potentially include water hyacinth, the next step was to discard all pixels outside the polygons intended to be analyzed by merging them with vegetation-index data. This way, pixels only within the polygons were kept. Then a threshold value representing vegetation within the water channels was defined for a binarization process where all pixels above it were classified as one and equal or below the threshold value, as zero. This binarization process lead to the creation of a new image where bright pixels represented water hyacinth, which also allowed us to calculate its overall occupation area for each water channel as well as another raster product with the vegetation-index values within the polygons. This way, with the inclusion of different periods, the multi-temporal analysis and the detection of annual and seasonal changes was possible.

### 2.4. Data Processing

Sentinel-2 MSI datasets were used to calculate the NDVI, which is widely used to analyze vegetation in different contexts. It uses NIR and red bands and is computed as shown in (1). Sentinel-2 MSI B8 (NIR) and B4 (red) were used to calculate the index. The same index was generated for the orthorectified UAV-based data in the three surveyed areas.

(1) $$\mathrm{NDVI}=\frac{\mathrm{NIR}-\mathrm{Red}}{\mathrm{NIR}+\mathrm{Red}}$$

A vector shapefile was created with the water channels intended to be analyzed (Figure 1). These data can be obtained through online platforms such as OpenStreetMap or from governmental entities. Moreover, it can be easily edited or created from scratch through photointerpretation of publicly available aerial imagery, such as Google Earth or Bing Maps, among others. In the context of this study, the data were downloaded from OpenStreetMap and then adjustments took place to reflect most parts of the water channels analyzed. The NDVI raster files of all dates (Table 1) were restrained to the polygon areas and binarized according to a threshold value which, in the case of this study, was selected from a UAV-Satellite data analysis (see Section 2.6). The threshold value can be obtained in three ways: (1) by georeferencing areas on the field where there are water hyacinth, and using that information to identify the corresponding pixels in the image and calibrating the value; (2) using photointerpretation of the remote sensing data (i.e., by observing the NDVI values in areas occupied by the invasive plant species), and then adapting/adjusting the threshold value to the data characteristics using a GIS; (3) analyzing the bimodal distribution of the histogram values within the areas to monitor.

### 2.5. Developed Tool

To automate most aspects of the methodology, a GIS open-source application was developed, named QIASdetection (QGIS Invasive Aquatic Species detection) [33]. It is operated in QGIS, a free and open-source GIS software, and the Python programming language and several application programming interfaces (APIs) were used to develop the main code. The framework Qt Designer was used to create and design the graphical user interface (GUI).

The application allows users to detect invasive species in aquatic ecosystems by analyzing the NDVI (already created or estimated from red and NIR bands). The input layers can be accessed from the QGIS canvas or from a user-specified directory. The user can define the NDVI filtering with two options: (1) by using a threshold value; or (2) by defining an interval by specifying minimum and maximum values. The application generates two outputs: (1) a raster identifying the invasive species (marked with a value of 1); and (2) a shapefile composed by descriptive statistics, such as the estimated area. The output files are stored in a folder defined by the user.

### 2.6. Data Analysis

To evaluate the water-hyacinth-detection accuracy when applying the methodology used in this study, two validation procedures were conducted using the acquired UAV-based data. One by conducting a pixel-wise comparison of the water hyacinth detection with manually digitized binary images of the UAV surveyed areas. The other assessment passed through the comparison of the obtained results (using the UAV-based NDVI) with the results obtained when applying a pixel-wise machine learning classification based on an RF [32] (the dataset is composed of the reflectance of the five orthorectified spectral bands acquired from the multispectral sensor onboard the UAV).

UAV data were also correlated with Sentinel-2 data for classification purposes. Due to differences in their spatial resolution—approximately 0.07 m for data acquired by UAV and 10 m for data from Sentinel-2—it is expected that some pixels may contain different elements. Therefore, to evaluate possible differences that may exist in the satellite data, the NDVI generated by the UAV data were resampled to the same spatial resolution as the NDVI generated with the Sentinel-2 data on 28 July 2021. A total of 100 pixels were selected that entirety cover water hyacinth areas and the correlation coefficient (R^2^) was evaluated.

The temporal monitoring of water hyacinth presence was conducted by analyzing each period regarding the monthly and annual patterns in its area and location, allowing us to monitor vegetative growth and/or decline over time. The relationship between the mean NDVI of the study area and water hyacinth cover percentage was subject to evaluation to understand the dynamics between these two parameters.

## 3. Results

### 3.1. Sentinel-2 and UAV Data Correlation

**Figure 2:**
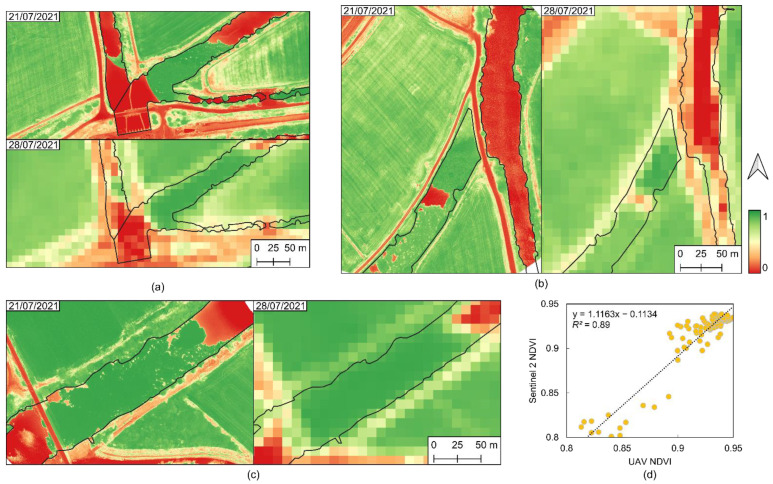
Normalized difference vegetation index (NDVI) of area 1 (**a**), area 2 (**b**) and area 3 (**c**) surveyed by the unmanned aerial vehicle (UAV) on 21 July 2021 and by Sentinel-2 on 28 July 2021, and (**d**) the linear correlation of water hyacinth NDVI pixels obtained from Sentinel-2 and UAV.

Despite the spatial resolution differences, the correlation between the UAV and Sentinel-2 NDVI data (Figure 2) had a good agreement between them with R^2^ = 0.89 (Figure 2d), demonstrating that there is no significant discrepancy in the values of both platforms and that they follow a similar spatial trend. When analyzing the data correlation obtained through the two platforms, satellite and UAV (Figure 2d), it was found that pixels with a high density of water hyacinth contained NDVI values around 0.8, but there may be pixels containing a lower density of plants. However, some disagreements may have occurred due to temporal changes. Thus, by considering the range of values in Figure 2d and the photointerpretation of the NDVI values on areas with water hyacinth (Figure 2a–c) it was decided to consider a threshold value of 0.75. This way, pixels above this value are considered to represent the invasive species under study.

### 3.2. Comparison of NDVI Thresholding and Machine Learning Performance

The validation conducted when considering all NDVI values above 0.75 in the three areas surveyed by the UAV (locations shown in Figure 1) enabled the comparison with the manually digitized masks representing water hyacinth. Area 3 showed the highest area of water hyacinth (above 9000 m^2^), followed by area 2 and area 1 with 5940 m^2^ and 5885 m^2^, respectively. The estimated water hyacinth coverage area for the three areas is presented in Table 2. A total of 598 m^2^ were estimated in excess when using the NDVI. On the other hand, the RF estimation resulted in a total underestimation area of 1312 m^2^.

**Figure 3:**
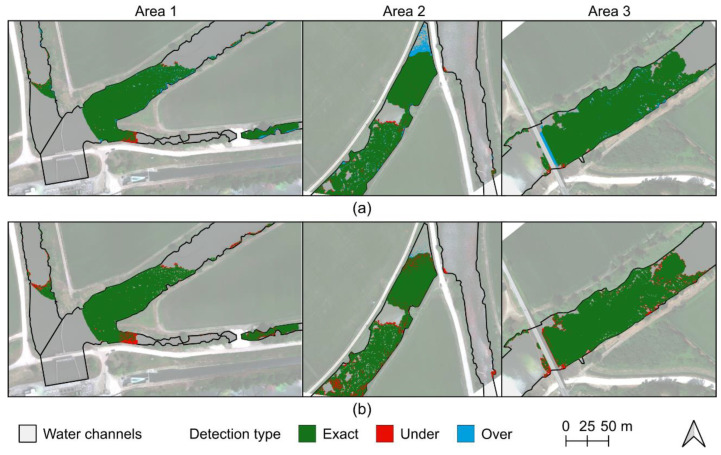
Water hyacinth classification results from normalized difference vegetation index (NDVI) thresholding (**a**) and from the random forest classifier (**b**) in the three areas surveyed by the unmanned aerial vehicle (UAV) on 21 July 2021.

When comparing the classification results of the evaluated methods, the NDVI thresholding approach shows a mean detection of 97.81% (97.51% in area 1, 97.12% in area 2, and 98.79% in area 3) while the RF classifier provided a mean detection rate of 93.31% (93.84% in area 1, 91.02% in area 2, and 95.08% in area 3). Figure 3 presents a visual representation of the correctly, under-, and over-detected pixels when compared with the manually digitized masks in both approaches. Both approaches present misclassifications in a small region of area 1, this being more noticeable in the RF classification. In area 2, another plant species was classified as water hyacinth when relying on the NDVI and a region of water hyacinth that had a considerable flower density was not detected by the RF algorithm. In area 3, some plants were not detected by both approaches, while when using NDVI there was a region located near the bridge that was over-detected.

### 3.3. Multi-Temporal Monitoring

**Figure 4:**
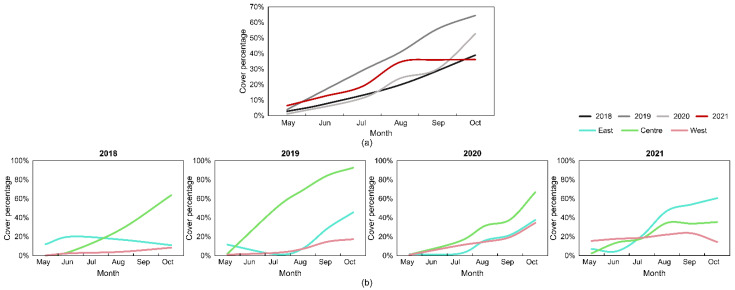
Water hyacinth temporal cover percentage evolution in each year, from May to October, for the whole study area (**a**) and in each analyzed water channel (**b**).

To analyze water hyacinth population growth and/or decline in the study area, Sentinel-2 time-series data were used (dates in Table 1). Water hyacinth distribution in different years analyzed (2018 to 2021) presented distinct behaviors, as observed in the overall occupation area shown in Table 3 and Figure 4a. In May 2018, about 3% (1.3 ha) of the channels showed signs of infestation by water hyacinth. This value rose to 20% (9.1 ha) in August, representing, in October, 39% of the total surface. In 2019, the infestation seems to have worsened. In May, the area covered by water hyacinth was 4% (+1% than 2018); in August the percentage of coverage was already higher than the maximum recorded in 2018, with 41% of the area analyzed being covered by water hyacinth; in October this rose to 64% (approximately 30 ha), which is the highest percentage of surface covered by water hyacinth recorded in the analyzed period. In 2020, only 1% (0.5 ha) of the surface was potentially covered by water hyacinth in May. In August this value rose to 24%, while in October it represented more than half (53%) of the entire analyzed surface. In May 2021, 6% of the area was estimated as being infested with water hyacinth, the highest value recorded for May. In August this value was 34% (approximately 16 ha), but in October there was a growth of only 2% compared to August (0% compared to September).

**Figure 5:**
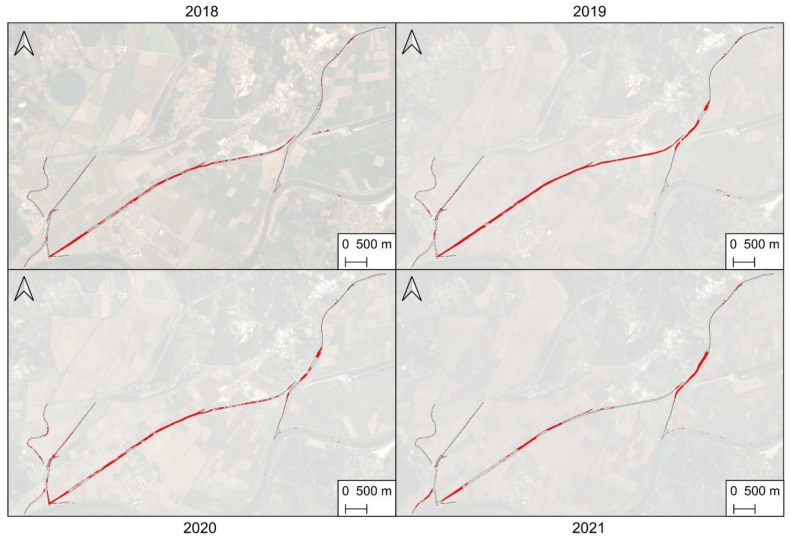
Spatial distribution of water hyacinth in the study area in October of 2018, 2019, 2020, and 2021.

**Figure A1:**
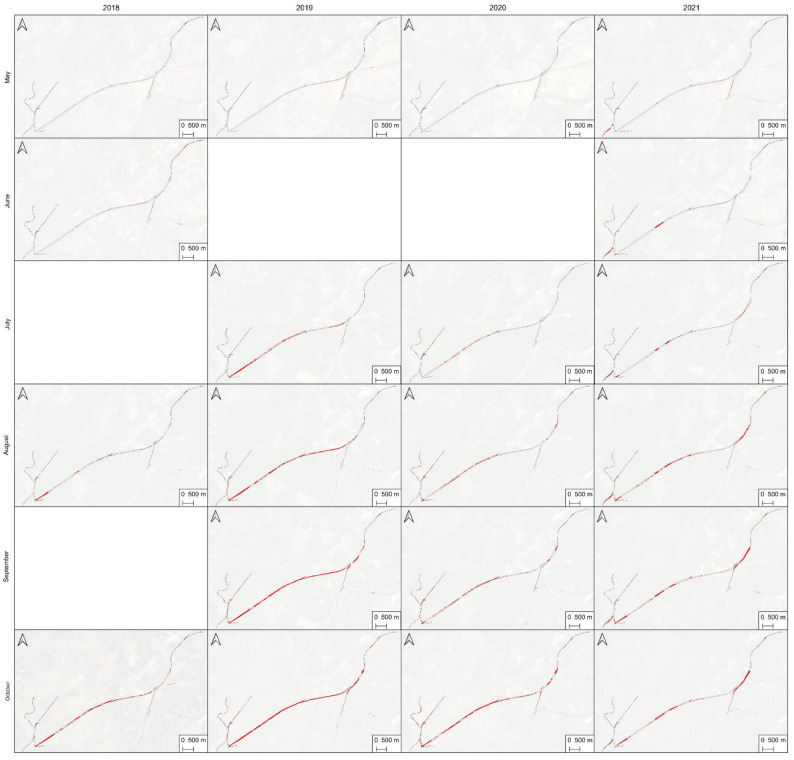
Water hyacinth spatial evolution in the study area between 2018 and 2021.

The contribution of each water channel to the temporal changes of water hyacinth cover were analyzed in the monitored period (cover values in Table 3, cover percentage in Figure 4b, and spatial distribution in Figure A1). It should be noted that in May of both 2018 and 2019, there was a higher percentage of water hyacinth in the east part. In the following months, the central channel showed a substantial growth in all years except 2021. As of October 2019, the central channel was almost entirely occupied by water hyacinth (distribution in Figure 5). In 2021, a decline was verified in the west and center water channels of the study area, yet there was an increase in the eastern channel.

**Figure 6:**
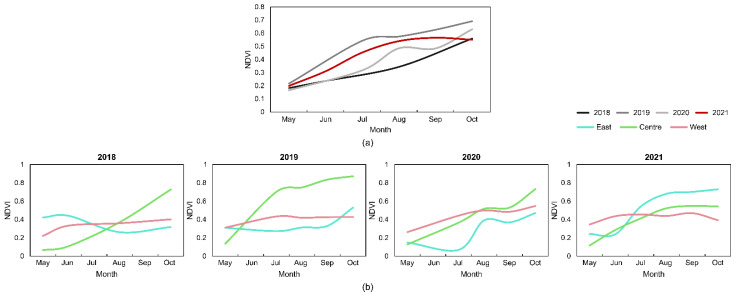
Mean normalized difference vegetation index (NDVI) temporal evolution in each year, from May to October, for the whole study area (**a**) and in each analyzed water channel (**b**).

Regarding the mean NDVI of the studied area (Figure 6a), there was a constant growth over time. May had the lowest values in the four years, approximately 0.2, with the lowest value observed in May 2020. On the other hand, October presented the highest mean NDVI values in 2018, 2019, and 2020 (respectively, 0.56, 0.69, and 0.63), while, in 2021, the highest value was reached in September (0.56). In June, the lower values were observed in 2018 and 2020 (0.24), followed by 2021 (0.31) and 2019 (0.38). The same trend was observed in July, August, and September 2018, presenting the lower mean values, followed by 2020 and 2021, and the higher mean values observed in 2019. Regarding the mean NDVI value per channel (Figure 6b), the central channel presented the lowest value in the four years (respectively, 0.07, 0.14, 13, and 0.12). On the other hand, the central channel presented the higher overall annual value in October 2018, 2019, and 2020, with a mean NDVI of 0.73, 0.87, and 0.73, respectively. In 2021, the highest mean NDVI value was recorded in October in the east channel (0.73).

**Figure 7:**
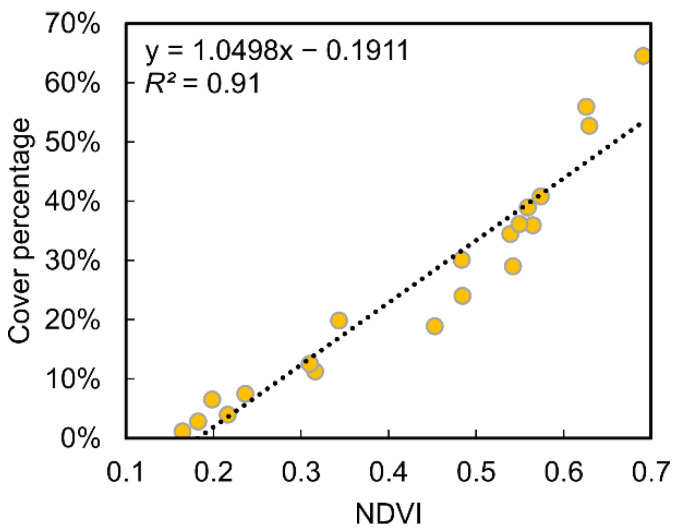
Linear correlation between water hyacinth cover percentage and mean normalized difference vegetation index (NDVI) of the water channels. Values from May to October 2018 to 2021.

The analysis of the relationship between the percentage of water hyacinth detected in the water channels and the mean NDVI is presented in Figure 7. A good data agreement was obtained with R^2^ = 0.91, meaning that there was a strong correlation between water hyacinth cover percentage and the mean NDVI value of the water channels. Moreover, similar trends can be verified in the multi-temporal analysis of both parameters (Figure 5 and Figure 6).

## 4. Discussion

Regarding the spatial NDVI correlation from UAV and Sentienl-2 (Figure 2d), in other studies that performed comparable assessments, similar distributions and correlations were observed among data from both platforms [14,34]. Nevertheless, some studies presented different correlation coefficients over time, depending on the vegetative development [35]. Concerning water hyacinth, John and Kavya [21] also found out that NDVI showed similar values to the ones obtained in this study. However, considering the greater spatial resolution that can be provided by UAV-based data, vegetation-filtering operations can be applied to the thousands of pixels that can fit in a single Sentinel-2 10 m^2^ cell [36,37]. On the other hand, the continuous temporal and spatial availability of Sentinel-2 data and the non-existent data-acquisition costs to end users promise a tremendous potential for certain applications. In this context, UAV data can be used to assist ground-truthing operations. Nevertheless, UAV data should be mainly considered when it is strictly required to obtain on-demand data for a specific time period and to collect validation data to complement satellite imagery [19].

The accuracy validation performed by using the UAV-based multispectral data revealed that the method tended to overestimate rather than underestimate (Table 2). The inverse was verified when applying the machine learning classification, which proved to be more prone to under-classifying water hyacinth. Moreover, the NDVI approach was able to correctly classify areas with a lower plant density or with a high presence of flowers, which the RF method had difficulty classifying (Figure 3). The over-detection cases can be perfectly justifiable as the NDVI provides higher values when there is a greater density of healthy green vegetation. The over-detection case verified in the third area was located under deep shadows (cast by the nearby bridge) which prevented to correct verification of the presence of water hyacinth when manually digitizing it, but it is perfectly viable that this specific estimation could be correct as there is a retention structure right after the bridge. Nevertheless, the developed tool [33] allows users to define a threshold range by specifying its maximum and minimum values, which can be explored in other contexts and also to decrease potential over-detection effects.

The water hyacinth multi-temporal analysis led to the exclusion of some months. In a preliminary analysis, and after consulting specialists with knowledge of the place under study, the winter and some spring months were disregarded, and the period between May and October of each year was selected for the final analysis, as it shows higher expansion rates during dry seasons of the year [18,23]. The decision to exclude the period between November and April was made due to the vegetative cycle of the species itself, which together with precipitation, and the consequent water-flow increase in winter and spring months, causes an almost immediate decrease in the amount of water hyacinth present in the analyzed sites. This is clearly evidenced in Gerardo and Lima [28], where part of the central water channel was analyzed, with a Sentinel-2 time-series that covered 2017 to 2021. The autumn corresponds to the senescence period and the gradual disappearance of water hyacinth [27]. Moreover, Janssens et al. [22] reported that the coverage of water hyacinth negatively correlates with rainfall and humidity increase.

When analyzing the multi-temporal monitoring results (Table 3, Figure 5 and Figure 7), it can be said that the growth of the infestation was progressive throughout 2018. This tendency worsened in 2019, where maximum values were registered in all analyzed months, except for May. In 2020, the lowest values were verified for the months of May and July, the coverage increased in August, but a slow growth was verified in September (+6% infestation, from 24% to 30%). The 2021 period, on the other hand, presented an expected trend in the first three months, with a sharp growth from July to August from 19% to 34%. However, unlike preceding years, an insignificant growth was observed in the subsequent months (September and October). The behavior observed in 2018 and mainly in 2019 may be related to the lack of combating the invasive species and the possibility of having favorable weather conditions for water hyacinth dispersion. The cover percentage in 2021 demonstrates that the imposed removal/mitigation actions on the study area had an effect, as the growth stagnated between August and October. Perhaps in 2020 the same type of operations had been carried out between August and September (see difference in Figure 5). The higher occupation in the central water channel (Table 3, Figure 6 and Figure A1) can be justified with the water-flow movement being from east to west, passing through the central channel and deriving in the same place where the west channel flows. The lower incidence of water hyacinth in 2021 was most likely related to the installation of retention structures and water-hyacinth-removal operations carried out in the field, showing a greater surface without infestation in the central channel (Figure 6) compared to previous years. Other authors also observed a decline period in water hyacinth between years, potentially due to the implemented control measures [20]. Moreover, different spatial distributions and coverage area were reported in various analyzed rivers and wetlands [19] or river parts presenting a higher peak in one of the monitored years [22,27], with a potential factor for this occurrence being drier climatic conditions.

The results reported in this study not only demonstrate that water hyacinth extent influences the NDVI value of the water bodies but also that NDVI is a suitable tool for its temporal monitoring. Nevertheless, the developed GIS application can also be used for vegetation segmentation in other contexts, such as forestry and agriculture. There are other approaches relying on machine learning for the detection of water hyacinth [19,22,25], but those require feature extraction and training stages, despite being possible to be directly applied in QGIS using plugins [9,32]. Moreover, machine learning classifiers can still provide false positives if water channels are not used to mask the results. Thus, the approach employed in this study is a rapid way to perform water hyacinth detection. NDVI thresholding proved to demonstrate good results with less complexity and at relatively lower computational costs, and it can also be implemented in different remote-sensing platforms and used in combination with ground-level sensors [17].

## 5. Conclusions

The content presented in this study summarizes part of the research work carried out for the detection and monitoring of water hyacinth in part of the Mondego River basin. A methodology was proposed to automatize most processes for water hyacinth detection using multispectral satellite data with 10 m spatial resolution. The image-processing techniques enabled us to filter out NDVI values that did not correspond to vegetation or were not within the region of interest, by using polygons to limit the classification only to aquatic bed zones.

If a fully automatic approach is intended, steps for automatically obtaining water bodies must be considered. One of the possibilities is to generate such masks through image processing techniques at low- or non-existence periods of water hyacinth incidence (winter or early spring). However, if the use of Sentinel-2 MSI data are considered, due to the nature of its spatial resolution and the reduced width of the water bodies, this type of approach can be inefficient, causing incorrect segmentation. The methodology is implemented in the form of a QGIS application. Therefore, a completely free approach is provided for monitoring water hyacinth presence in any area of the globe using Sentinel-2 MSI data or by using high-spatial-resolution UAV-based data. Thus, entities responsible for water hyacinth monitoring and control can use the proposed methodology in a fast and intuitive manner, helping to support decision making in mitigating/controlling invasive aquatic species.

As a future work, water hyacinth samples may be collected to correlate with the remotely sensed data to enable us to study the viability of directly estimating its biomass through vegetation indices from remotely sensed multispectral data. The continuous monitoring of the study area should continue to be performed to assess whether the results observed in 2021 are repeated or even decline, in order to assess the effectiveness of the applied mitigation measures.

## Figures and Tables

**Table 1:** Date (year, month, and day) of the Level-2A Sentinel-2 in the grid T29TNE.

Month/Year	2018	2019	2020	2021
January	—	10	05	—
February	—	14	19	28
March	—	11	10	20
April	—	20	—	04
May	05	05	24	19
June	19	—	—	23
July	—	14	18	28
August	18	13	22	17
September	—	12	01	21
October	02	22	11	19
November	—	—	—	—
December	31	—	—	—

**Table 2:** Water hyacinth detected area (m^2^) type (correct, over, and under) when using the normalized difference vegetation index (NDVI) and when applying a random forest (RF) in the three areas surveyed by the unmanned aerial vehicle.

Area No.	Approach	Detection Type (m^2^)			Estimated Area (m^2^)	Digitized Area (m^2^)
		Correct	Over	Under		
1	NDVI	5738	250	147	5988	5885
	RF	5522	2	362	5524	
2	NDVI	5769	425	171	6194	5940
	RF	5406	32	534	5438	
3	NDVI	9038	351	111	9389	9149
	RF	8699	0	450	8699	

**Table 3:** Estimated water hyacinth surface occupation (in hectares) from the analyzed periods, from May 2018 until October 2021.

Year	Month	East	Center	West	Total
2018	May	1.3	0.0	0.0	1.3
	June	2.1	1.1	0.3	3.5
	August	1.8	7.0	0.4	9.1
	October	1.2	15.9	0.9	18.0
2019	May	1.2	0.5	0.1	1.8
	July	0.1	12.9	0.3	13.4
	August	0.8	17.3	0.8	18.8
	September	3.0	21.2	1.6	25.8
	October	4.7	23.2	1.9	29.8
2020	May	0.2	0.3	0.1	0.5
	July	0.2	3.8	1.2	5.2
	August	1.7	7.9	1.6	11.1
	September	2.3	9.5	2.1	13.9
	October	3.9	16.7	3.7	24.3
2021	May	0.7	0.6	1.7	3.0
	June	0.5	3.4	1.9	5.8
	July	2.2	4.5	2.0	8.7
	August	4.9	8.6	2.4	15.9
	September	5.6	8.4	2.5	16.6
Total surface (ha)		6.3	8.9	1.5	16.7

## Data Availability

The data that support the findings of this study are available from the corresponding author upon reasonable request. The GIS application code is available at https://github.com/liaduarte/QIASdetection, accessed on 20 October 2022.

## Appendix A

Figure A1 presents the generated water hyacinth distribution maps in the monitored months (when data are available) between 2018 and 2021.
